# Efficacy and Safety of Aldose Reductase Inhibitor for the Treatment of Diabetic Cardiovascular Autonomic Neuropathy: Systematic Review and Meta-Analysis

**DOI:** 10.1371/journal.pone.0087096

**Published:** 2014-02-12

**Authors:** Xin Hu, Shengbing Li, Gangyi Yang, Hua Liu, Guenther Boden, Ling Li

**Affiliations:** 1 Key Laboratory of Diagnostic Medicine (Ministry of Education) and Department of Clinical Biochemistry, College of Laboratory Medicine, Chongqing Medical University, Chongqing, China; 2 Department of Endocrinology, the Second Affiliated Hospital, Chongqing Medical University, Chongqing, China; 3 Department of Pediatrics, University of Mississippi Medical Center, Jackson, Mississippi, United States of America; 4 The Division of Endocrinology/Diabetes/Metabolism and the Clinical Research Center, Temple University School of Medicine, Philadelphia, Pennsylvania, United States of America; Iran University of Medical Sciences, Iran (Islamic Republic of)

## Abstract

**Background:**

Aldose reductase inhibitors (ARIs) can block the metabolism of the polyol pathway, and have been used to slow or reverse the progression of diabetic cardiovascular autonomic neuropathy (DCAN). The purpose of this study was to review the effectiveness and safety of ARIs in the treatment of DCAN as determined by five cardiac autonomic neuropathy function tests.

**Methods:**

CENTRAL, MEDLINE, EMBASE, Scopus databases (inception to May 2012) were searched to identify randomized controlled trials (RCTs) and non-randomized controlled trials (non-RCTs) investigating ARIs for the treatment of DCAN with an English-language restriction. The data were analyzed using RevMan 5.0, and the heterogeneity between the trials was evaluated using the Cochrane's Q-test as well as the I^2^ test. The type of model (random or fixed) used for analysis was based on heterogeneity. Weighted mean differences (WMD) with 95% confidence intervals (CI) were computed for the five cardiac automatic neuropathy function tests to evaluate the effects.

**Results:**

Ten articles met the prerequisites for this review. Analysis of the results showed that ARIs significantly improved function in at least three of the five automatic neuropathy tests, including the resting heart rate variation coefficients (WMD = 0.25, 95%CI 0.02 to 0.48, P = 0.040); the 30∶15 ratio (WMD = 0.06, 95%CI 0.01 to 0.10, P = 0.010) and the postural systolic blood pressure change (WMD = −5.94, 95%CI −7.31 to −4.57, P = 0.001). The expiration/inspiration ratio showed a marginally significant benefit (WMD = 0.05, 95%CI 0.00 to 0.09, P = 0.040). Glycaemic control was not significantly affected by ARIs. Adverse effects of ARIs except for Tolerestat were minimal.

**Conclusions:**

Based on these results, we conclude that ARIs could ameliorate cardiac automatic neuropathy especially mild or asymptomatic DCAN but need further investigation.

## Introduction

Diabetes mellitus (DM) is becoming a world-wide problem with more people being affected each year. Cardiovascular autonomic neuropathy (CAN), a common diabetic complication, can result in arrhythmia, silent myocardial infarction, heart failure, and sudden death [1]–[4]. Many studies have shown an association between CAN and increased risk of mortality in individuals with diabetes [5]. To improve the poor prognosis and quality of life for these patients, early detection and therapeutic interventions are needed.

The etiology of diabetic neuropathy has thus far remained uncertain. Multiple factors have been implicated including endoneural ischemia, hypoxia, accumulation of glycated proteins, disorders of polyol metabolism, absence of nerve growth factors, disturbance of axonal transport as well as autoimmune damage [1], [3]–[4], [6]–[9]. However, the disorders of polyol metabolism are regarded as the major problem. Hyperglycemia activates the intracellular polyol pathway causing accumulation of sorbitol. Increased levels of cellular sorbitol lead to myoinositol deficiency, decreases in protein kinase C and Na/K-ATPase activity and change in NAD/NADH ratios. This results in cellular water and electrolyte imbalance and oxidative injury. Aldose reductase inhibitors (ARIs) block the rate-limiting enzyme of the polyol pathway, decrease the accumulation of sorbitol and improve nerve function [10], [11]. Based on these results, ARIs have been proposed as potential therapy for diabetic neuropathy.

A number of studies have demonstrated the effectiveness and safety of ARIs as therapy for diabetic peripheral neuropathy (DPN), but few have assessed the effectiveness of ARIs as therapy for diabetic cardiovascular autonomic neuropathy (DCAN). A review including 13 trials with ARIs as therapy for DPN was reported in 2007 [12], but DCAN was not included in that review. In addition, conflicting results of ARIs as therapy for DCAN have been reported in several trials [13]–[26]. We, therefore, conducted a meta-analysis of controlled clinical trials which investigated the role of ARIs in the treatment and prevention of DCAN.

## Methods

### 1.1 Data Sources and Searches

We searched the PUBMED/MEDLINE databases, the EMBASE, the Scopus and the Cochrane Collaboration databases (from inception to May 2012) for randomized placebo-controlled clinical trials (RCTs) and non-randomized controlled trials (non-RCTs) using ARIs for the prevention of DCAN in subjects with no known history of other diseases which might interfere with cardiovascular reflex test results. The search terms were: ‘aldose reductase inhibitors’, ‘aldehyde reductase inhibitors’, ‘Alrestatin’, ‘Sorbinil’, ‘Epalrestat’, ‘Statil’, ‘Tolrestat’, ‘Ponalrestat’, ‘Fidalrestat’, ‘Zenarestat’ or ‘Zopolrestat’ and ‘diabetic cardiovascular autonomic neuropathy’ or ‘diabetic neuropathy’. The search was limited to human studies published in English using cardiovascular reflex tests. We used the same strategy to search the EMBASE and CENTRAL databases. In addition, we searched pertinent references from the included articles. The U.S. Food and Drug Administration (FDA), European Medicines Agency Web sites and some pharmaceutical companies' databases were searched for unpublished trials. We also attempted to contact the authors of relevant studies to retrieve missing data.

### 1.2 Study Selection

The inclusion criteria employed were: 1) a RCT or non-RCT design; 2) use of ARIs with recommended doses and specifications as treatment for DCAN; 3) a treatment period of at least three months; 4) an outcome defined as change of cardiovascular autonomic nerve function, measured by at least one cardiovascular reflex test, 5) sufficient data for the statistical analysis.

Included were subjects who were at least 18 years old, and in whom the diagnosis of DM and DCAN was clearly stated and in whom other diseases such as liver or renal failure, thyroid dysfunction, alcoholism, nutritional deficiency, malignant disease, ischemic heart disease, heart failure, valvular heart disease, and major cardiac arrhythmias which might confuse cardiovascular reflex test results were excluded. Patients receiving digitalis, anticholinergics, sympathomimetics, beta-blockers, antiarrhythmic drugs, or other drugs affecting heart-rate variability were also excluded.

### 1.3 Data Extraction and Quality Assessment

Two of the authors independently reviewed the contents of 589 abstracts and full-text articles, identified through the search strategies to determine whether they met the eligibility criteria. Data extraction was carried out independently by the same authors. From each study, the following data were collected: 1) author, year, population location, trial design; 2) type of ARIs used, dose, frequency and duration of administration, presence of placebo controls; 3) Patient number, age, duration of DM and DCAN; 4) Anthropometrics and laboratory tests (baseline and end points), results of cardiovascular autonomic reflex tests, glycated hemoglobin (HbA_1c_) and adverse effects. When relevant clinical data were missing in the published articles, we attempted to contact the corresponding author in order to obtain additional information. All discrepancies between the two authors were resolved by discussion with a third author.

We developed a methodological quality assessment tool based on the criteria published by Jadad [27]. It focused on the following categories awarding a maximum of five points to each study: randomization procedures; allocation concealment; double blinded assessment of participants, personnel, and outcome assessors; a description of withdrawals or dropouts with follow-up rates or dropout rates and the use of intention-to-treat analysis. The quality of the included randomized clinical trials was assessed by three categories ranging from A (high quality) to C (low quality). A total score of each trial was obtained by following these criteria. Any disagreement regarding study quality was resolved by discussion among the authors. Studies were not excluded on the basis of poor quality as there was insufficient evidence for a relationship between criteria to measure internal validity and research outcomes [28].

### 1.4 Outcome Measures

The primary outcome was a change of cardiovascular autonomic nerve function as determined by a set of cardiovascular reflex tests which were recommended as diagnostic tests for DCAN by the American Diabetic Association and the American Neurologic Academy [29], [30]. These tests have good sensitivity, specificity, and reproducibility and are noninvasive, safe, well standardized, and easily performed. However, the Valsalva maneuver should not be performed on patients with proliferative retinopathy, because it may increase the risk of intraocular haemorrhage or lens dislocation [31], [32]. Secondary outcomes were the change of HbA_1c_ levels (a measure of glycaemic control) and any adverse effects.

We selected the following five cardiovascular autonomic reflex tests for this review: (i) coefficient of variation of R-R intervals (CV_R-R_): After a 10 min rest in supine position, 100–150 consecutive heart beats are recorded on an continuous ECG and the coefficient of RR variation is calculated as the standard deviation of R-R intervals/the mean value of R-R intervals. (ii) expiration/inspiration ratio (E/I ratio): Subjects breathe deeply in and out 6 times with continuous ECG monitoring. The longest R-R interval during expiration (E) and the shortest R-R interval during inspiration (I) are used to calculate the E/I ratio. (iii) 30∶15 ratio: Patients rest for 15–30 min in a supine position, and then get up. The heart rate (HR) on ECG after changing from the supine to upright position is recorded. The R-R interval ratio at the HR nadir around beat 30 after standing up divided by the HR peak near beat 15 is expressed as 30∶15 ratio. (iv)Valsalva ratio: Ratio of the longest R-R interval after the Valsalva maneuver to the shortest interval during the maneuver. (v) Postural systolic blood pressure change (postural SBP change): Change in systolic blood pressure beginning 30 seconds after assuming the upright posture. The tests from (i)–(iii) mainly reflect parasympathetic function, (v) indicates principally sympathetic function, and (iv) reflects sympathetic and parasympathetic functions [29]–[34].

### 1.5 Statistical Analysis

The data were processed on a personal computer, and statistical calculations and graphs were performed using the Review Manager (RevMan) software package (Version 5.1.0 for Windows the Nordic Cochrane Centre, Copenhagen, Denmark). Procedure and reporting followed QUOROM guidelines [35]. Weighted mean differences (WMD) with 95% confidence intervals (CIs) were calculated for changes from baseline in ARIs or placebo groups. We recorded the mean value of baseline and follow-up levels of the five autonomic tests for the ARIs and controlled groups and the standard error (SEM) or standard deviation (SD) of each mean. Then we converted the value of SEM to SD with the formula from the Cochrane Handbook for Systematic Reviews of Interventions: 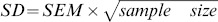 [28]. Mean net change values were calculated as the difference (ARIs minus placebo) in the changes (follow-up minus baseline) of the mean values. If the SD of the difference for each group was not reported, it was estimated through the method of Follmann et al, assuming a correlation coefficient of 0.5 between initial and final values. The SD of the mean net change values was calculated using the following formula: 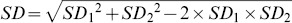, SD_1_: the SD of baseline mean; SD_2_: the SD of follow-up mean; R: the correlation coefficient (R = 0.5) [28], [36]. Each study was weighted for its variance in order to pool the data for overall effect size. The variances were calculated using CIs, P-values and, *t*-statistics or individual variances for the two groups. Estimates of effect, WMD, were pooled using the Inverse variance method. For each outcome, a forest plot was created that illustrates both study-specific and pooled WMD with 95% CIs. All tests were two-sided with statistical significance when P-value≤0.05, if not otherwise specified.

Heterogeneity between the trials in each meta-analysis was evaluated using Cochrane's Q-test and the inconsistency index I^2^ [37], [38]. When I^2^>50% or Q-test X^2^ P<0.05, we determined there was a significant heterogeneity, and the random effects model was used, while in the absence of significant heterogeneity between the included trials, the fixed effects model was used [39], [40]. Subset analysis was performed to explore the possible sources of heterogeneity in: (I) type of ARIs, (II) duration of diabetes mellitus, (III) duration of intervention, (IV) the level of HbA_1_c, if data from more than two trials were available. A symmetric funnel plot of each outcome was graphically examined to assess the potential publication bias by constructing it with variance plotted against the corresponding effect sizes. Egger's test, carried out with Stata 10 (StataCorp, College Station, Tex), was also used to evaluate the publication bias.

## Results

### 2.1 Search Results and Eligible Studies

**Figure 1:**
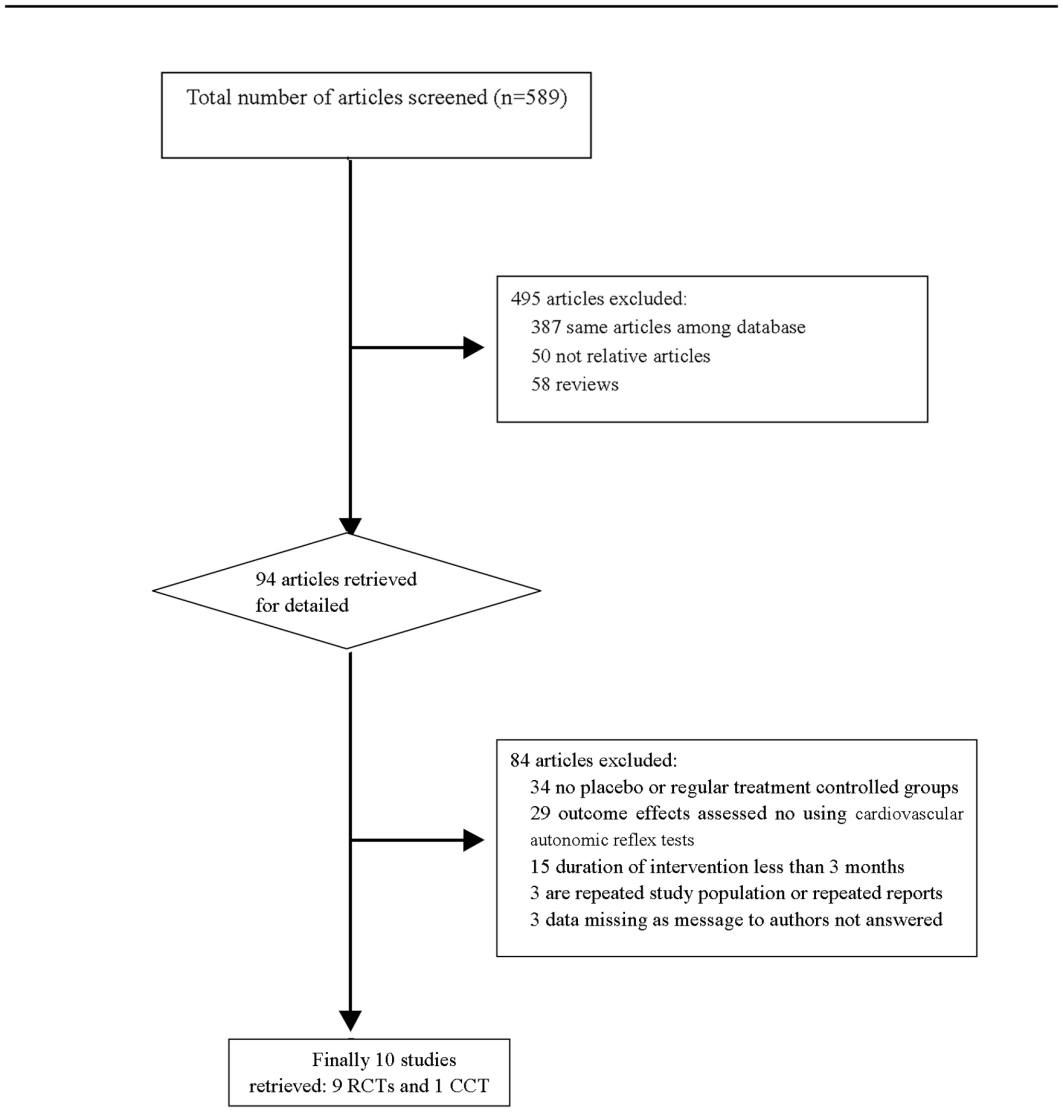
Flowchart of search strategy and results.

A total of 589 citations were retrieved. Ten of these met the study selection criteria. The results of the literature search are summarized in **Figure 1**. Nine studies were RCTs, one was non-RCT. Four trials used epalrestat, four used ponalrestat, and two used tolrestat. The ARIs were given orally with their recommended doses, and compared with a placebo. The characteristics of these studies are shown in **Table 1**.

**Table 1:** Summary of General information and Methodological quality of included studies.

First Author and publication year	Population Location	Research design	Intervention	Duration of intervention (month)	Number of patients (ARIs/Placebo)	Age (ARIs/Placebo) (Mean±SD)	Duration of diabetes (Year) (Mean±SD)	HbA_1c_ (%) (ARIs/Placebo)		Primary outcomes of DCAN (ARIs/Placebo) (Mean±SD)
								Baseline (Mean±SD)	Follow-up (Mean±SD)	
Goto Y et al 1995 [13]	Japan	RCT	Epalrestat 50 mg po tid	3	54/61	56.5±0.9/57.4±0.9	NR	NR	NR	CV_R-R_ 1.3±7.009/0.3±1.353
Hotta N et al 2006 [17]	Japan	RCT	Epalrestat 50 mg po tid	36	134/159	61.5±9.1/61.5±9.8	12.4±8.0/13.3±8.9	7.0±1.0/7.1±1.0	7.3±1.3/7.1±1.0	CV_R-R_ −0.17±1.27/−0.26±1.31
Ikeda T et al 1998 [18]	Japan	non-RCT	Epalrestat 50 mg po tid	36	22/43	64.8±7.8/66.5±9.8	18.3±5.6/13.3±8.9	8.58±1.96/8.64±1.72	8.47±1.73/8.54±1.67	CV_R-R_ 0.37±1.009/−0.21±0.709
Nakayama M et al 2001 [19]	Japan	RCT	Epalrestat 50 mg po tid	6	15/13	60.9±1.8/66.5±9.8	14.1±1.6/11.2±2.3	7.71±0.85/7.39±0.12	7.69±1.05/7.39±1.05	CV_R-R_ 0.56±1.494/−0.19±2.03; E/I ratio 0.1±0.216/−0.1±0.2; Postural SBP change −8±16.476/0.1±8.839
Faes TJ et al 1993 [16]	Netherlands	RCT	Ponalrestat 600 mg po qd	6	15/12	47.6±1.9/47.6±1.9	17.7±1.8/17.7±1.8	8.2±0.3/9.1±0.4	7.4±0.3/8.8±0.6	E/I ratio 0.025±0.078/0.048±0.073; 30∶15 ratio0.045±0.202/0.01±0.122; Valsalva ratio 0.152±0.51/−0.03±0.157
Sundkvist G et al 1992 [20]	England, Norway, Sweden	RCT	Ponalrestat 600 mg po qd	18	182/77	45±12/48±11	20±10/21±9	8.79±2.25/8.79±2.17	9.2±2.12/9.01±1.85	E/I ratio −0.01±0.185/0±0.141; 30∶15 ratio 0±0.175/−0.03±0.157; Valsalva ratio −0.01±0.345/−0.05±0.061
Gill JS et al 1990 [22]	England	RCT	Ponalrestat 600 mg po qd	4	17/13	55.6±8.9/58.6±5.6	12.2±6.8/12.7±7.1	NR	NR	Postural SBP change−3.4±15.357/1.7±12.1
Ziegler D et al 1991 [21]	German	RCT	Ponalrestat 600 mg po qd	12	39/21	53.8±1.3/46.9±2.5	15.7±1.3/15.3±1.5	9.5±1.87/9.1±1.36	9.2±1.25/9.5±1.83	CV_R-R_ 0.05±1.268/−0.12±1.59; E/I ratio 0.01±0.108/−0.05±0.137
Giugliano D et al 1993 [14]	Italy	RCT	Tolrestat 200 mg po qd	13	25/20	55.3±6.2/56.8±6.4	9.8±3.5/9.5±3.4	8.2±1.1/8.4±1.1	NR	E/I ratio 0.03±0.046/−0.01±0.05; 30∶15 ratio 0.03±0.046/−0.01±0.05; Valsalva ratio 0.04±0.155/−0.01±0.036; Postural SBP change−5.9±8.278/−1±8.305
Giugliano D et al 1995 [15]	Italy	RCT	Tolrestat 200 mg po qd	13	29/28	56.5±6/55±8	6.2±2.7/5.6±2.8	7.0±0.5/7.2±0.5	NR	E/I ratio 0.06±0.06/−0.04±0.056; 30∶15 ratio 0.06±0.061/−0.05±0.061; Valsalva ratio 0±0.075/0.01±0.122; Postural SBP change−6±2.598/0±3

*RCT = randomized controlled trial; non-RCT = non-randomized controlled trial; po = per os; tid = three times a day; qd = once a day. NR = not report. SD =  standard deviation.

### 2.2 Quality Assessment of Included Studies

Each study was reviewed by two of the authors to evaluate characteristics of methodological quality in the aforementioned aspects and each characteristic was graded as adequate, inadequate and unclear or degree not reported. The details of the quality assessment of the included trials are summarized in **Table 2**.

**Table 2:** Summary of Methodological Quality of Included Studies.

First Author and publication year	Randomized generator	Allocation Concealment	Blinding	Lost to Follow-Up	Data analysis	Scores
Goto Y et al 1995 [13]	A	B	A	A	APT	4
Hotta N et al 2006 [17]	A	A	C	A	APT	3
Ikeda T et al 1998 [18]	C	C	C	B	APT	1
Nakayama M et al 2001 [19]	A	B	A	A	APT	4
Ziegler D et al 1991 [21]	A	B	A	A	APT	4
Faes TJ et al 1993 [16]	A	B	A	A	APT	4
Sundkvist G et al 1992 [20]	A	B	A	A	APT	4
Gill JS et al 1990 [22]	A	B	A	B	APT	3
Giugliano D et al 1993 [14]	A	B	A	A	APT	4
Giugliano D et al 1995 [15]	A	B	A	A	APT	4

*A: adequate; **B**: inadequate; **C**: unclear or not report; **APT:** all patients treated, defined as those who received at least one dose of study treatment and who had both a baseline and at least a follow-up measurement.

### 2.3 Primary Outcomes

The results of the statistical analyses are shown in **Table 3**.

**Table 3:** Summary of the comparisons about the five automatic tests and HbA_1c_.

Outcome or subgroup title	No. of studies	No. of participants	Statistical method	Effect size (WMD, 95%CI)
1.CV_R-R_	5	561	Mean Difference (IV, Fixed, 95% CI)	0.25 [0.02,0.48]
2.E/I ratio	6	476	Mean Difference (IV, Random, 95% CI)	0.05 [0.00,0.09]
2.1.Ponalrestat alone	3	346	Mean Difference (IV, Random, 95% CI)	0.00 [−0.03,0.03]
2.2.HbA1c<8.0% alone	2	85	Mean Difference (IV, Random, 95% CI)	0.10 [0.07,0.13]
3.30∶15 ratio	4	388	Mean Difference (IV, Random, 95% CI)	0.06 [0.01,0.10]
3.1.DM>9.5years	3	331	Mean Difference (IV, Random, 95% CI)	0.04 [0.01,0.06]
4. Valsalva ratio	4	388	Mean Difference (IV, Fixed, 95% CI)	0.03 [−0.01,0.06]
5. Postural SBP change	4	160	Mean Difference (IV, Fixed, 95% CI)	−5.94 [−7.31,−4.57]
6. HbA_1c_	6	889	Mean Difference (IV, Random, 95% CI)	−0.11 [−0.42, 0.19]

*IV = Inverse variance; Random = random effects model; Fixed = fixed effects model; WMD = weighted mean difference; CI = confidence interval.

#### 2.3.1 Coefficient of variation of silent R-R intervals (CVR-R)

**Figure 2:**
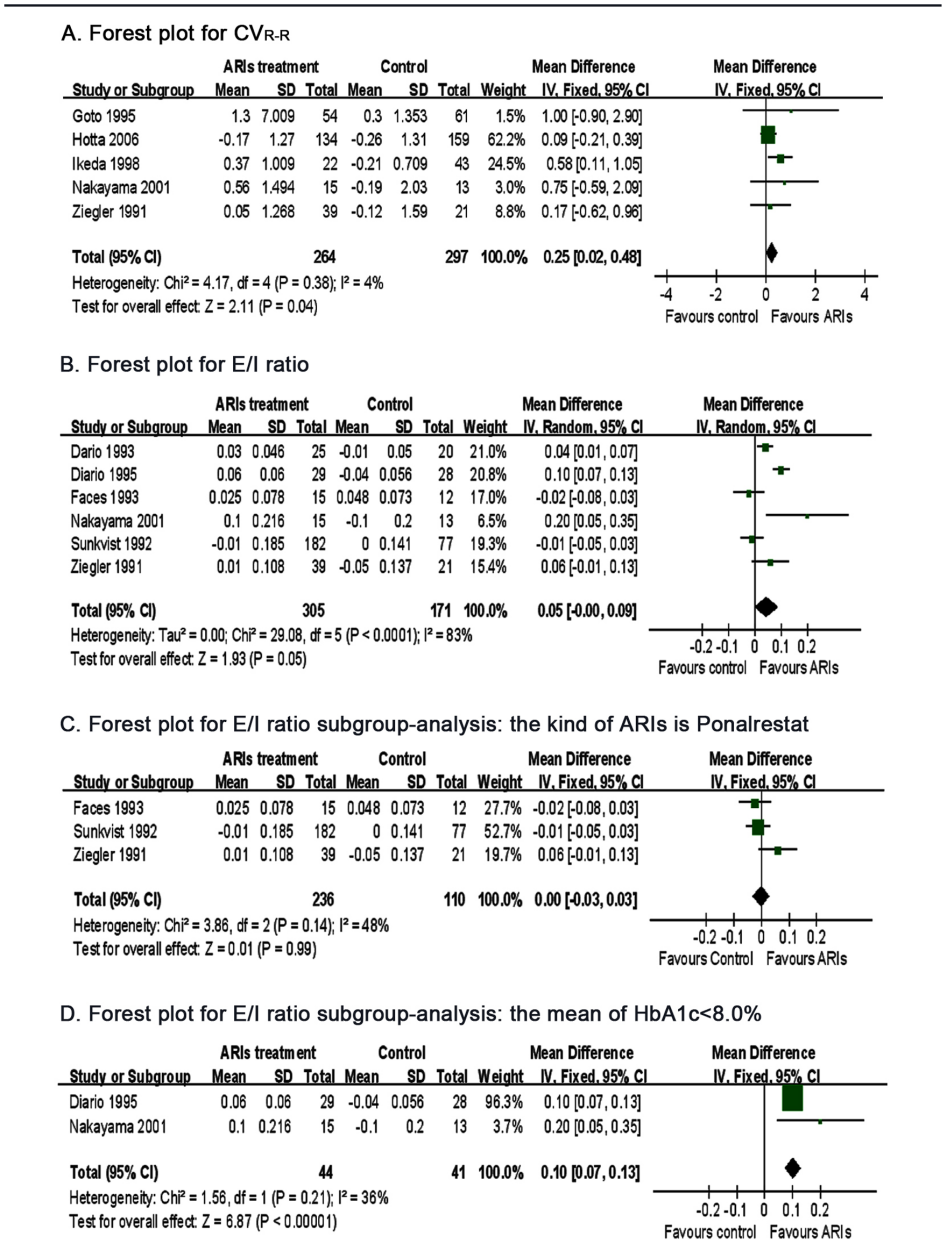
Results of meta-analysis of the five cardiovascular autonomic reflex tests and HbA_1c_.

Five trials analyzed the effect of ARIs on the CV_R-R_ [13], [17]–[19], [21]. There was no significant heterogeneity (P = 0.380, I^2^ = 4%) in these studies which were analyzed by the fixed effects model. CV_R-R_ is used as an index reflecting cardiovascular parasympathetic nerve function. In general, CV_R-R_ is lower in patients with T2DM than in healthy subjects. CV_R-R_ was significantly increased in ARIs-treated patients (WMD = 0.25, 95%CI 0.02 to 0.48, P = 0.040) (**Fig. 2A**), indicating that the DCAN was improved by ARIs therapy.

#### 2.3.2 Expiration/inspiration ratio (E/I ratio)

Six trials analyzed the effect of ARIs on the E/I ratio [14]–[16], [18]–[20]. Because there was significant heterogeneity in these studies (I^2^ = 83%), a random effects model was used. The E/I ratio was also used to assess the cardiovascular parasympathetic nerve function. The E/I ratio was slightly increased in patients treated with ARIs compared (WMD = 0.05, 95%CI 0.00 to 0.09, P = 0.040) (**Fig. 2B**), suggesting that ARIs may improve automatic nerve function.

We performed a subgroup analysis to find the source of the high heterogeneity. Included in the subgroup analysis were the type of ARIs and HbA_1_c levels. Low heterogeneity was found in the Ponalrestat group (I^2^ = 43%) suggesting that there was no significant effect of Ponalrestat on the E/I ratio (WMD = 0, 95%CI −0.03 to 0.03, P = 0.990) (**Fig. 2C**). There was also low heterogeneity in the subgroup with HbA1c<8.0% (I^2^ = 36%). A statistical difference in the E/I ratio was found between ARIs and control groups (WMD = 0.01, 95%CI 0.07 to 0.13, P = 0.000) (**Fig. 2D**), suggesting that ARIs, except Ponalrestat, may be more effective in patients with better glycaemic control.

#### 2.3.3 30∶15 ratio

**Figure 3:**
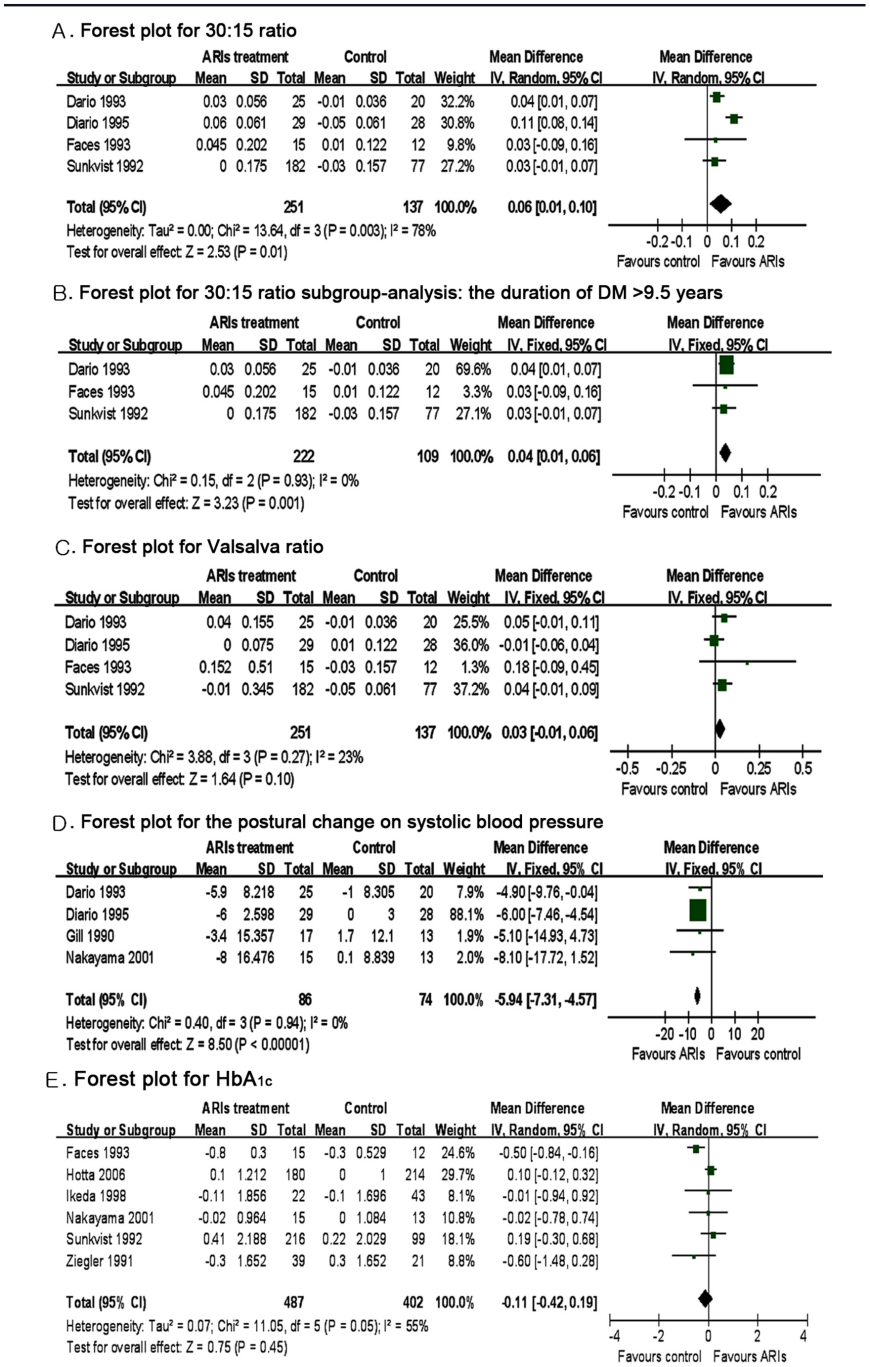
Results of meta-analysis of the five cardiovascular autonomic reflex tests and HbA_1c_—Continued.

The 30∶15 ratio was assessed in four studies [14]–[16], [20]. A random effects model was used to uncover heterogeneity in these studies (Q-test, P = 0.003, I^2^ = 78%). The 30∶15 ratio was higher in the ARIs-treated than the placebo-treated patients (WMD = 0.06, 95%CI 0.01 to 0.10, P = 0.009) (**Fig. 3A**), indicating that ARIs therapy improved the 30∶15 ratio, an index for DCAN.

To find the source of the high heterogeneity, we conducted a subgroup analysis to examine the effects of duration of diabetes on the 30∶15 ratio. There was no heterogeneity in the subgroup with duration of diabetes>9.5 years (I^2^ = 0%), demonstrating that the duration of diabetes may be a confounder in this analysis. There was a statistical difference in the 30∶15 ratio (WMD = 0.04, 95%CI 0.01 to 0.06, P = 0.001) (**Fig. 3B**) between ARIs-treated and non-treated patients.

#### 2.3.4 Valsalva ratio

The Valsalva ratio was assessed in four studies [14]–[16], [20]. A fixed effect model was used due to lack of significant heterogeneity (Q-test, P = 0.270, I^2^ = 23%). The results revealed that there was no significant difference in the Valsalva ratio between ARIs and placebo treated patients (WMD = 0.03, 95%CI −0.01 to 0.06, P = 0.100) (**Fig. 3C**).

#### 2.3.5 The effect of postural SBP change

The effect of postural SBP change was assessed in four studies [14], [15], [19], [22]. As there was no heterogeneity in these studies (Q-test, I^2^ = 0%, P = 0.940), the fixed effects model was used for analyses. A significant difference was observed between ARIs and placebo treatment (WMD = −5.94, 95%CI −7.31 to −4.57, P = 0.000) (**Fig. 3D**), suggesting that ARIs treatment may improve orthostatic hypotension.

### 2.4 Glycaemic Control

None of the trials included show significant differences in the HbA1c levels between ARIs and placebo treatments. Only six trials contained sufficient data for statistical analysis. The results showed that there was no statistical significant difference between the ARIs and controls (WMD = −0.11, 95%CI −0.42 to 0.19, P = 0.450) (**Fig. 3E**). Thus, it appears that the use of ARIs would not influence the regular management of glycemia.

### 2.5 Adverse Effects

Adverse effects were reported with varying degrees of detail and the data were insufficient to perform statistical comparisons. Epalrestat was reported to increase liver enzymes and cause nausea and diarrhea in a few patients in one of our studies. Discontinuation of the drug resulted in recovery. Another study reported a decrease in hemoglobin, erythrocyte and lymphocyte counts. Ponalrestat was well tolerated in the included studies, but decreases in hemoglobin, red blood cell count and lymphocyte count are reported in other studies. Tolrestat was generally well tolerated. One of the included studies reported reductions in erythrocyte indices and total protein, and slightly increased levels of alanine aminotransferase, alkaline phosphatase and gamma glutamyl transferase. Other publications reported that Tolrestat was associated with elevation of liver enzymes, dizziness, reduced blood pressure and fatal hepatic necrosis [41].

### 2.6 Publication Bias Assessment

**Figure 4:**
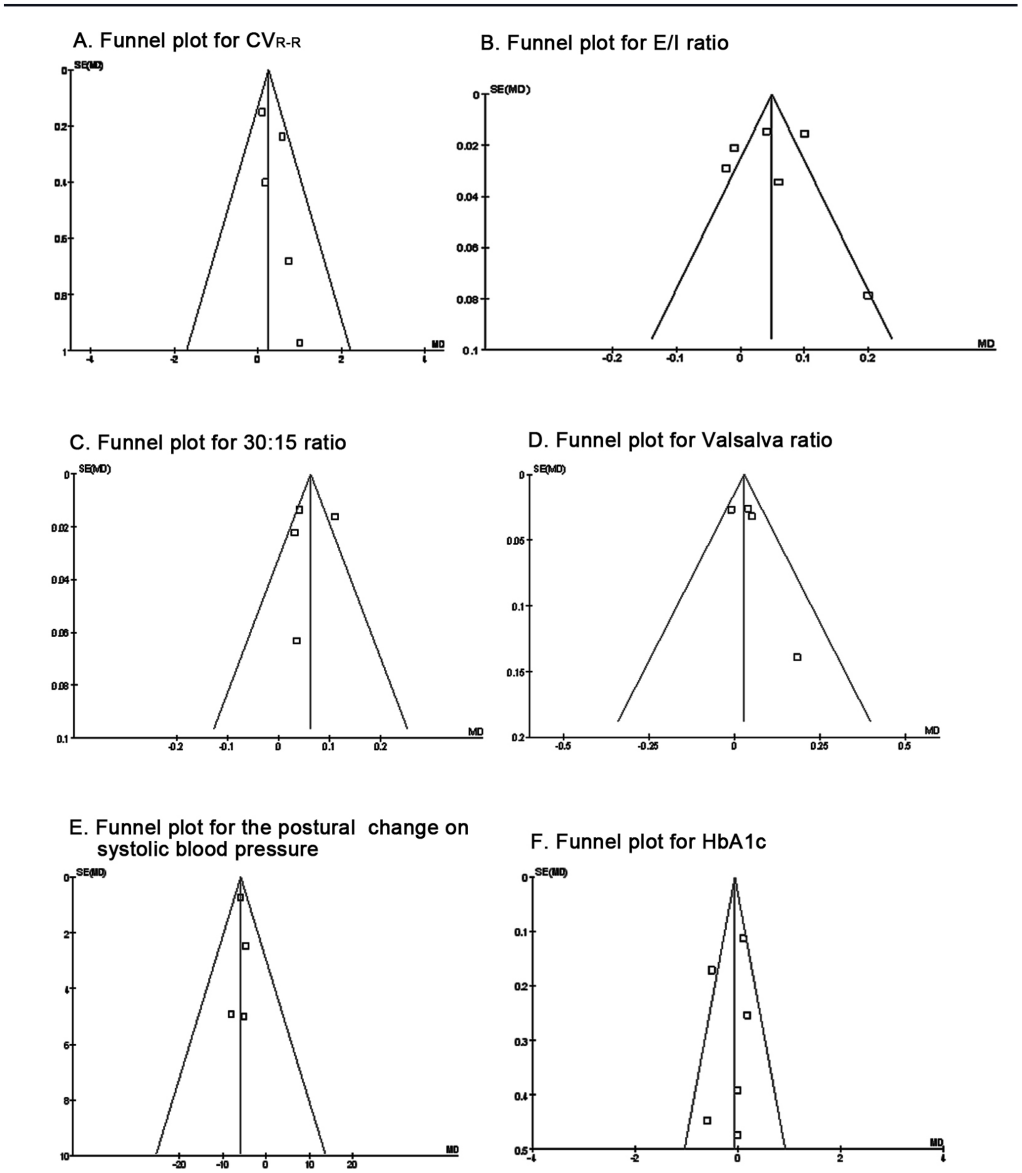
Funnel plot of the five cardiovascular autonomic reflex tests and HbA_1c_.

A symmetric funnel plot of each outcome was graphically examined to assess the potential publication bias in **Figure 4**. Egger's test was also done for each outcome. All the P-values of the five cardiovascular reflex tests and HbA_1c_ were greater than 0.05, and their 95% CI of intercept included zero in Egger's publication bias tests (**Table 4**). These results indicate that funnel plots in this meta- analysis were symmetrical without publication bias.

**Table 4:** Egger's test for publication bias.

Bias of Egger's test	CV_R-R_	E/I ratio	30∶15 ratio	Valsalva ratio	postural change on SBP	HbA_1c_
P-value	0.182	0.194	0.368	0.225	0.765	0.077
95% CI	−1.514, 5.129	−2.896, 10.337	−10.007, 17.349	−2.569, 6.043	−61.549, 72.169	−4.591, 0.368

## Discussion

Our meta-analysis is the first study that systematically reviews the efficacy and safety of ARIs for the treatment of DCAN. Previous meta-analyses have focused on the effects of ARIs on DPN. Cardiovascular autonomic reflex tests were used to assess these therapeutic trials [42], [43]. Cardiac radionuclide imaging, microneurography and other new examination techniques have recently been used to quantify cardiac autonomic nerve functions in various cardiac diseases, including DCAN, and have been reported to be more sensitive than indirect cardiovascular autonomic reflex tests. However, they are not used in the majority of clinical trials because of cost or invasiveness [42]–[45]. The meta-analysis showed that ARIs therapy can significantly improve at least three of the five tests, including the CV_R-R_, the 30∶15 ratio and postural SBP changes. All the drugs showed a marginally significant benefit to the E/I ratio, and a significant benefit was associated with ARIs from most of the drugs except Ponalrestat. The Valsalva ratio was not different between ARIs and placebo (P = 0.10). The CV_R-R_, the E/I ratio and the 30∶15 ratio all reflect parasympathetic nerve function, whereas postural SBP changes reflect sympathetic nerve function. Only the Valsalva ratio reflects both parasympathetic and sympathetic nerve function. Perhaps that was why the Valsalva ratio showed no difference in the two groups. In addition, the type of ARIs, HbA_1_c level and the duration of diabetes were considered as possible sources for heterogeneity in the results of the five cardiovascular autonomic reflex tests. We found that Ponalrestat did not improve the E/I ratio in the subgroup analysis. We also found a statistical difference in the E/I ratio in the subgroup with HbA1c<8.0%, suggesting that ARIs therapy may be more effective in patients with better glycaemic control. Subgroup analysis also demonstrated that the duration of diabetes may be a confounder in the analysis of the 30∶15 ratio, suggesting that ARIs may be more effective in the early course of DCAN. The HbA_1c_ was not affected by ARIs in any of the studies and thus ARIs did not seem to interfere with the action of hypoglycemic agents in diabetic patients. As for the adverse effects of ARIs, they were generally minimal in these studies, discontinuation of the drug resulted in recovery. No severe adverse effects such as death, be life-threating, hospitalization, persistent or significant disability or incapacity, congenital anomaly or birth defect were reported, which means ARIs are safe and sound for the clinical use to the treatment of DCAN.

A meta-analysis, performed by Chalk et al in 2007, reported no significant benefit of ARIs on DPN [12]. However, most subjects in these studies had severe symptoms including pain and numbness, indicating advanced damage to nerve function which was probably irreversible. In support, another study also has shown that ARIs could improve 3-Iodobenzylguanidine uptake, measured by cardiac radionuclide imaging, and heart rate variability in diabetic patients with early, but not with advanced DCAN [42]. Furthermore, the selection criteria of subjects, trials and primary outcomes were different between Chalk's and our study, and DCAN was not evaluated in Chalk's meta-analysis. Cardiac autonomic nerves may be impaired earlier than peripheral nerves. Peripheral nerves are myelinated while autonomic postganglionic nerves are unmyelinated. These differences may be a reason why cardiac autonomic nerves may respond more favorably to ARIs treatment than peripheral nerves [20]. We therefore believe that ARIs can play a beneficial role in the treatment DCAN. Hyperglycemia, plays a key role in the activation of polyol pathway and the pathogenesis of DCAN in type 1 DM (T1DM) and type 2 DM (T2DM). The 10 controlled clinical trials in our review contained patients with T1DM and T2DM. Thus, we believe that the effects of ARIs therapy are similar in patients with T1DM and T2DM.

Our meta-analysis has limitations: 1) in some studies, it was difficult to clarify details of methodology and results. 2) We excluded some studies that did not use the five reflex tests which may have led to publication bias. 3) Only Asian and Europe populations and three ARIs were included in our meta-analysis. 4) Because we limited our search to articles written in English, we may have missed studies published in other languages. 5) Endpoints such as silent myocardial ischemia and sudden death were not analyzed which made it difficult to assess the long-term effect of ARIs.

## Conclusion

The results of this meta-analysis provide statistical evidence that ARIs have some benefits when used as treatment for DCAN, while having no influence on glycaemic control and having few adverse effects. The results also suggested that ARIs are more effective in mild and asymptomatic DCAN than its advanced cases. However, more and long-term randomized comparative studies with new ARIs are needed to define the effects of ARIs on DCAN.

## Supporting Information

Checklist S1 **PRISMA checklist.**
(DOC)
